# Parapneumonic pleurisy in patients with community bacterial pneumonia

**DOI:** 10.1186/1471-2334-14-S7-P52

**Published:** 2014-10-15

**Authors:** Daniela Ristea, Dan Hurezeanu, Livia Dragonu, Maria Balan, Carmen Canciovici, Mioara Cotulbea

**Affiliations:** 1 “Victor Babeş” Clinical Hospital of Infectious Diseases and Pneumology, Craiova, Romania; 2 University of Medicine and Pharmacy Craiova, Romania

## Background

Parapneumonic pleurisy has a variable incidence on patients with community bacterial pneumonia, worsening the disease evolution. The purpose of the study is to emphasize the frequency of parapneumonic pleurisy occurrence correlated with some contributing factors.

## Methods

A retrospective study has been performed on a group of 87 patients with community bacterial pneumonia (average age of 49.7 years, M/F proportion, FINE seriousness score of 72-127). The diagnosis has been established clinically, radiologically, and through complex bacteriological and serological examinations.

## Results

The parapneumonic pleurisy has been diagnosed during the first days after onset (1-5 days) in 64 of the patients, for the rest after 6-7 days of evolution. Bacteriological tests identified *Streptococcus pneumoniae* from sputum in 12 cases, *Haemophilus influenzae* in 2 cases. Eight patients have been found with high titer IgG antibodies for *Chlamydia pneumoniae* and 2 cases with IgM antibodies for *Chlamydia pneumoniae*. The virulence of the bacterial bases has been similar on the patients with and without parapneumonic pleurisy. The contributing and aggravating factors were the smoking in 68% of cases, diabetes mellitus in 47%, hypo-proteinemia in 23%, cardiac failure in 2%, ischemic heart in 1.5% of patients.

## Conclusion

The parapneumonic pleurisy is both an aggravating factor and a severity indicator for the evolution of community acquired pneumonia. The occurrence of parapneumonic pleurisy is encouraged by the metabolic and physical disorder provoked by smoking and the related co-morbidities.

